# Comparison of Arch Index Derived from Optical Pedography and Barometric Platform in Children: A Method Agreement Study

**DOI:** 10.3390/jfmk11020228

**Published:** 2026-06-03

**Authors:** Miloslav Gajdoš, Jakub Čuj, Katarína Hnatová, Wioletta Mikuľáková, Lucia Demjanovič Kendrová

**Affiliations:** 1 Department of Physiotherapy, Faculty of Health Care, University of Prešov in Prešov, 080 01 Prešov, Slovakia; jakub.cuj@unipo.sk (J.Č.); katarina.hnatova@unipo.sk (K.H.); wioletta.mikulakova@unipo.sk (W.M.); lucia.kendrova@unipo.sk (L.D.K.); 2 Rehabilitation and Podiatry Clinic Fyziopoint, 080 01 Prešov, Slovakia

**Keywords:** arch index, plantar pressure, pedobarography, optical pedography, method comparison, Bland–Altman analysis, pediatric foot

## Abstract

**Objectives:** The Arch Index (AI) is commonly used to assess medial longitudinal arch morphology; however, values obtained using different measurement technologies may not be interchangeable. This study aimed to compare AI values derived from optical pedography and a barometric platform during bilateral static stance assessment in children and to evaluate their agreement. **Methods:** Thirty-eight healthy children aged 5–10 years underwent standardized bilateral static foot assessment. AI was calculated using identical segmentation and formula for both systems. Paired t-tests, Pearson correlation, intraclass correlation coefficient, and Bland–Altman analysis were used to assess agreement between methods. **Results:** Optical pedography produced significantly higher AI values than barometric assessment for both the left (0.284 ± 0.055 vs. 0.188 ± 0.092) and right foot (0.286 ± 0.048 vs. 0.169 ± 0.072; *p* < 0.001). Agreement between methods was moderate (ICC = 0.494–0.581), with wide limits of agreement. Inter-method differences increased with age. **Conclusions:** AI values obtained from optical pedography and barometric platforms are not interchangeable in children. Consistent use of a single measurement technology is recommended in pediatric assessment to avoid misinterpretation of developmental changes.

## 1. Introduction

The medial longitudinal arch plays a fundamental role in load distribution, shock absorption, and postural stability during weight-bearing activities [1,2]. In children, the plantar architecture undergoes progressive development, particularly between 5 and 10 years of age, when structural maturation of the arch and gradual reduction in the plantar fat pad occur. Previous studies have emphasized that plantar arch development during childhood is influenced by both structural maturation and progressive changes in plantar soft tissue properties [3,4,5,6]. Variability in arch morphology during this period is considered physiological; however, inaccurate interpretation may lead to overdiagnosis or inappropriate therapeutic interventions [7]. The Arch Index (AI), defined as the ratio of midfoot area to total footprint area excluding the toes, is a widely used measure of medial longitudinal arch morphology. Although the Arch Index has traditionally been derived from footprint-based methods, several studies have also applied plantar pressure assessment systems for foot type classification and plantar arch evaluation [8,9]. However, pressure-based measurements may be influenced by factors such as sensor resolution, pressure thresholds, and plantar loading characteristics, which can affect direct comparability with footprint-derived measurements [9,10,11]. Several footprint-based parameters have been proposed to assess medial longitudinal arch morphology, including the Chippaux–Smirak Index, Staheli Index, and Clarke’s angle. Nevertheless, the Arch Index remains widely applied due to its simplicity, reproducibility, and applicability across different measurement systems. Originally introduced by Cavanagh and Rodgers [12], it has been widely applied in both clinical practice and research settings [13,14]. With technological advancements, AI calculation has shifted from ink footprints to digital systems, primarily optical pedography and barometric platforms. Although both systems apply the same three-region segmentation principle and Arch Index formula, their detection principles differ substantially. Optical systems measure geometric contact area independent of pressure magnitude, whereas barometric platforms register only regions exceeding sensor pressure thresholds [10,11]. In pediatric populations, these technological differences may be particularly relevant due to increased compliance of soft tissues and lower static midfoot pressures [3,15]. To date, limited evidence exists regarding the interchangeability of AI values obtained from these technologies in children [10,13]. Reliable agreement between different measurement systems is essential in biomechanical assessment. Therefore, the aim of this study was to compare Arch Index values obtained using optical pedography and a barometric platform during static bilateral stance in children aged 5–10 years and to evaluate the degree of agreement between methods. We hypothesized that Arch Index values obtained from optical pedography and barometric platforms would differ systematically and would not demonstrate sufficient agreement to be considered interchangeable.

## 2. Materials and Methods

### 2.1. Participants

Thirty-eight healthy children aged 5–10 years participated in this study. Data were collected between January 2025 and January 2026. All examinations were conducted at the Rehabilitation Clinic Fyziopoint during preventive musculoskeletal assessments.

Inclusion criteria were as follows: age between 5 and 10 years, absence of diagnosed orthopedic or neurological disorders, no history of lower limb surgery, and no acute musculoskeletal pain at the time of examination.

The study was approved by the Ethics Committee of the University of Prešov (approval No. 05/2024). Written informed consent was obtained from the parents or legal guardians of all participants. The study was conducted in accordance with the Declaration of Helsinki.

Although no formal a priori power analysis was performed, the sample size was considered adequate for this exploratory method-comparison study and was comparable to those used in similar previously published pediatric plantar morphology and method-comparison studies.

### 2.2. Measurement Protocol

**Figure 1:**
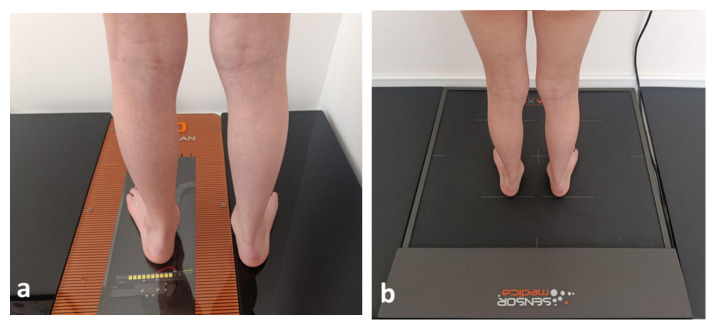
Representative measurement setup during static bilateral stance assessment using (**a**) optical pedography and (**b**) barometric platform.

All measurements were performed during static bilateral stance under standardized conditions. Participants stood barefoot on the measurement surface with arms relaxed alongside the body and gaze directed forward. A single 5 s recording was obtained for each device. Repeated measurements were not averaged in order to maintain a simple and clinically applicable assessment protocol and to minimize fatigue and postural variability in pediatric participants during repeated static stance trials. The reported Arch Index values were derived from these single recordings. Measurements were performed sequentially using the following systems: Podoscan 3D (Sensor Medica, Guidonia Montecelio, Italy) equipped with freeStep software (version 1.X) and a barometric platform (Sensor Medica, Guidonia Montecelio, Italy). Both systems were used according to the manufacturers’ recommendations. Both optical pedography and barometric platform assessment are commonly used methods for plantar surface evaluation and have previously demonstrated acceptable reliability and validity in foot morphology and plantar assessment studies [8,9]. Representative measurement setup during static bilateral stance assessment is shown in Figure 1.

### 2.3. Arch Index Calculation

**Figure 2:**
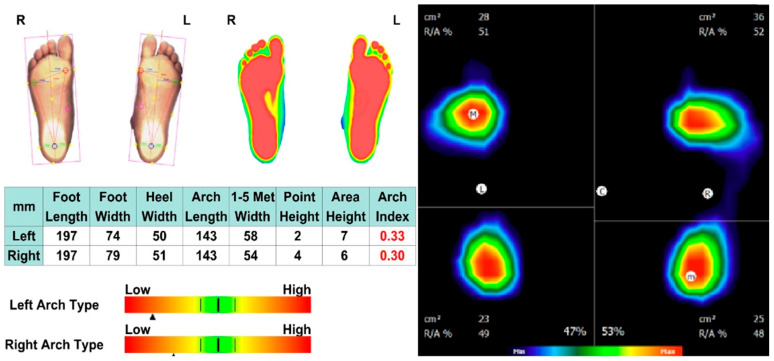
Representative plantar outputs and Arch Index measurements obtained from optical pedography and barometric platform assessment.

**Figure 3:**
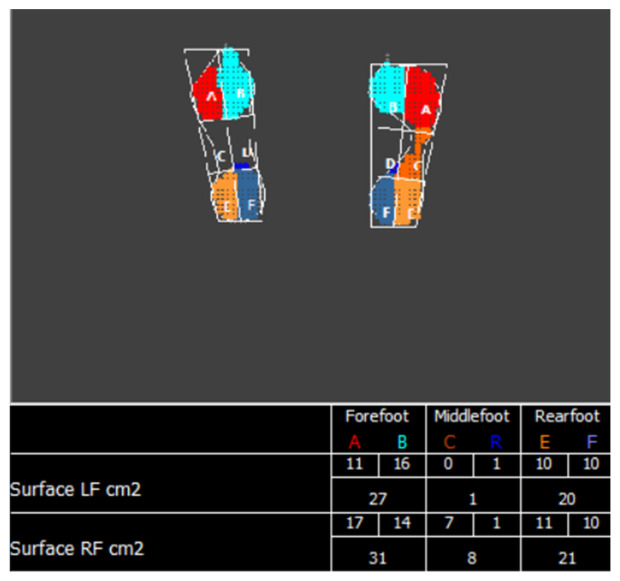
Representative plantar segmentation and contact area measurements used for Arch Index calculation from barometric platform assessment.

For both systems, the plantar surface was divided into three equal regions along the longitudinal axis (rearfoot, midfoot, and forefoot), excluding the toes. The Arch Index was calculated according to the method described by Cavanagh and Rodgers [12] as the ratio of midfoot area to the sum of rearfoot, midfoot, and forefoot areas. The same segmentation principles and mathematical formula were applied for both devices. Values were obtained from the respective software systems. For the barometric platform, Arch Index values were calculated using the complete plantar contact area detected by the pressure sensors, including all low-pressure regions registered by the software. Areas without measurable plantar pressure were not included in the midfoot contact area used for Arch Index calculation. Therefore, differences between optical and barometric Arch Index values may reflect the distinction between geometric plantar contact detected by optical systems and biomechanically loaded plantar contact detected by pressure-based systems. Representative plantar outputs and Arch Index measurements obtained from both systems are shown in Figure 2. The segmentation procedure and contact area measurements used for Arch Index calculation are illustrated in Figure 3.

### 2.4. Statistical Analysis

Statistical analyses were performed using Python (version 3.11) with the SciPy and Statsmodels libraries. Descriptive statistics were calculated for all variables. Normality of data distribution was assessed using the Shapiro–Wilk test. Paired *t*-tests were used to compare AI values between methods. Pearson correlation coefficients were calculated to assess associations. Agreement between methods was evaluated using the intraclass correlation coefficient (ICC, two-way random effects model, single measures—ICC(2,1)) as described by Shrout and Fleiss [16], and Bland–Altman analysis with 95% limits of agreement [17]. Effect size was calculated using Cohen’s d for paired samples. Multivariable linear regression was performed with inter-method difference (Podoscan−Barometry) as the dependent variable and age, body mass index, and sex as independent variables. Statistical significance was set at *p* < 0.05. Height and weight were not included in the regression model due to their collinearity with body mass index, which was selected as the representative anthropometric variable.

## 3. Results

Thirty-eight children (age range: 5–10 years) were included. Descriptive characteristics of the study population are presented in Table 1.

Comparison between optical pedography and barometric platform measurements is summarized in Table 2.

Multivariable regression analysis results are presented in Table 3.

**Table 3:** Multivariable regression analysis of inter-method differences.

Predictor	Left Foot β	Left *p*	Right Foot β	Right *p*
Age	0.016	0.008	0.012	0.010
Body mass index	0.004	0.412	0.003	0.531
Sex	−0.011	0.287	−0.009	0.344

Arch Index values measured by optical pedography were significantly higher than those obtained by barometric assessment for both feet (*p* < 0.001).

**Figure 4:**
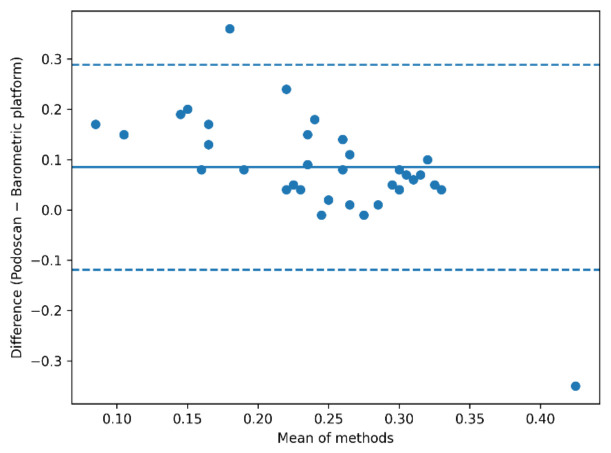
Bland–Altman plot for the left foot showing agreement between optical pedography and barometric platform. The solid line represents the mean difference (bias), while the dashed lines represent the upper and lower 95% limits of agreement.

For the left foot, the mean paired difference was 0.097 ± 0.077. Bland–Altman analysis demonstrated limits of agreement from −0.053 to 0.247 (Figure 4). Pearson correlation demonstrated a moderate association (r = 0.562, *p* < 0.001), and the intraclass correlation coefficient indicated moderate agreement (ICC(2,1) = 0.494).

**Figure 5:**
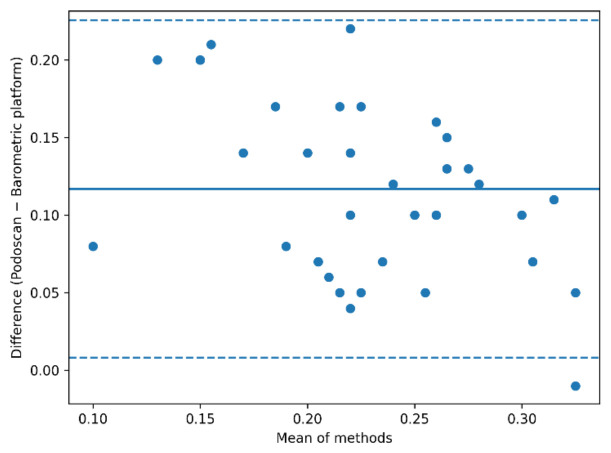
Bland–Altman plot for the right foot showing agreement between optical pedography and the barometric platform. The solid line represents the mean difference (bias), while the dashed lines represent the upper and lower 95% limits of agreement.

For the right foot, the mean paired difference was 0.117 ± 0.056, with limits of agreement from 0.007 to 0.227 (Figure 5). Pearson correlation demonstrated a moderate association (r = 0.628, *p* < 0.001), and the intraclass correlation coefficient indicated moderate agreement (ICC(2,1) = 0.581).

**Figure 6:**
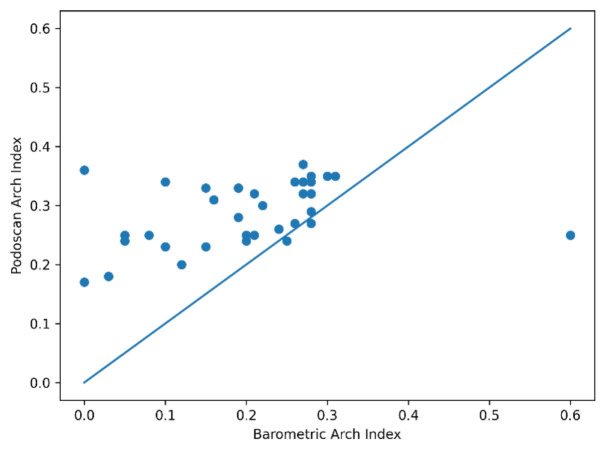
Scatter plot showing the correlation between Arch Index values obtained from optical pedography and the barometric platform for the left foot. The solid line represents the linear regression trend line.

**Figure 7:**
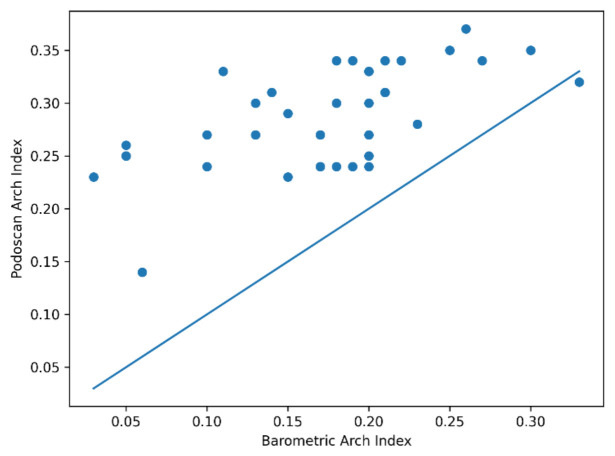
Scatter plot showing the correlation between Arch Index values obtained from optical pedography and the barometric platform for the right foot. The solid line represents the linear regression trend line.

Scatter plots illustrating correlations between methods are shown in Figure 6 and Figure 7.

Inter-method difference was positively correlated with age for both left (r = 0.435, *p* = 0.006) and right foot (r = 0.406, *p* = 0.011). In multivariable regression analysis adjusting for body mass index and sex, age remained independently associated with inter-method difference (right foot: β = 0.012, *p* = 0.010; left foot: β = 0.016, *p* = 0.008). Body mass index and sex were not independently associated with inter-method differences in either foot (*p* > 0.05).

No significant associations were observed between body mass index or sex and Arch Index values obtained from either measurement system (*p* > 0.05).

## 4. Discussion

This study demonstrates a systematic and clinically relevant bias between optical pedography and barometric assessment of the Arch Index in children aged 5–10 years. Optical pedography consistently produced higher AI values than the barometric platform for both feet. These findings are consistent with previous studies reporting that pressure-based systems may produce lower midfoot contact area estimates compared with geometric footprint methods [10,18,19].

Although the correlation between methods was moderate, the agreement analysis revealed only moderate ICC values and relatively wide limits of agreement, indicating that the two technologies cannot be considered interchangeable at the individual level. From a biomechanical perspective, pressure-based systems may provide a more functionally relevant representation of plantar loading because only regions generating measurable plantar pressure contribute to the calculated contact area. It is well established that correlation does not imply agreement, and method comparison studies require specific agreement analyses such as Bland–Altman evaluation [17,20]. Slight differences observed between left and right foot agreement parameters may reflect natural functional asymmetry and variability in pediatric postural control during static stance. Similar variability in plantar loading characteristics has been reported in developing children [21]. Our results reinforce the distinction between statistical association and true measurement interchangeability.

The systematic positive bias observed in the present study can be explained by differences in detection principles. Optical pedography measures geometric contact area independent of pressure magnitude, whereas barometric platforms detect only regions exceeding a specific pressure threshold [10,22]. Additionally, barometric systems may differ in spatial resolution and sensor sensitivity, which can further influence plantar contact detection and Arch Index calculation. In a static stance, midfoot loading in children is typically lower than in adults, which may lead to underestimation of the midfoot area when pressure-based systems are used [15,21].

As demonstrated by the regression analysis and correlation analysis in the Results section, inter-method differences increased significantly with age for both feet, suggesting that technological bias becomes more pronounced during arch maturation. Developmental changes in plantar soft tissue thickness, particularly reduction in the plantar fat pad, and progressive structural stabilization of the medial longitudinal arch may alter pressure distribution patterns with age [3,4,23]. As tissue stiffness increases and load transfer becomes more localized, differences between geometric and pressure-based detection may become more pronounced.

From a biomechanical perspective, the Arch Index represents an area-based morphological parameter rather than a force-based variable. Consequently, pressure-based systems may systematically exclude low-pressure contact regions that are nevertheless anatomically relevant [10,19]. This distinction is particularly important in pediatric populations, where plantar soft tissues are more compliant and arch development is ongoing [15,23].

Clinically, these findings highlight the importance of methodological consistency in pediatric screening and longitudinal monitoring. Switching between measurement technologies may produce artificial changes in Arch Index values that do not reflect true morphological adaptation. Similar concerns have been reported in other method-comparison studies in biomechanical research [20,22].

From a clinical perspective, these findings emphasize the need for device-specific reference values in pediatric foot assessment.

Future studies should investigate whether comparable discrepancies exist under dynamic conditions during gait, where load magnitude and temporal characteristics differ from static stance [2,24,25]. Additionally, further work is needed to determine whether calibration adjustments or standardized pressure thresholds could reduce inter-method bias in pediatric assessments.

### Limitations

Several limitations of the present study should be acknowledged. First, measurements were performed during static bilateral stance; therefore, the findings primarily apply to static assessment and cannot be directly extrapolated to dynamic gait conditions.

Second, a single 5 s recording was obtained for each device. Although this approach reflects common clinical practice, it may not fully capture intra-individual variability.

Third, the sample size, while sufficient to detect statistically significant inter-method differences, was limited to healthy children without diagnosed foot pathology. Consequently, the findings may not be directly generalizable to clinical populations. The sample size was considered adequate for detecting moderate-to-large inter-method differences based on the exploratory design of the study and the statistically significant effects observed in the agreement analyses.

Finally, inter-session and inter-rater reliability were not evaluated in this study. In addition, numerical values of individual plantar regions were available from the barometric platform software, but were not available for all participants from the optical pedography system. Direct comparison of regional plantar contact areas between systems was therefore not possible because the optical pedography software exported only final Arch Index values and did not provide numerical segmental area data for all participants. Consequently, regional plantar contact areas could not be statistically compared between the two technologies.

## 5. Conclusions

Arch Index values obtained from optical pedography and barometric platforms are not interchangeable in children aged 5–10 years. Optical systems systematically yield higher values, and inter-method differences increase with age. Consistent use of a single measurement technology is recommended in pediatric clinical and research applications to ensure methodological consistency and avoid misinterpretation of developmental changes.

## Figures and Tables

**Table 1:** Participant characteristics (n = 38).

Variable	Mean ± SD
Age (years)	7.4 ± 1.6
Height (cm)	126.4 ± 11.5
Weight (kg)	26.8 ± 6.3
Body mass index (kg/m^2^)	16.6 ± 1.9
Sex (M/F)	17/21

**Table 2:** Comparison of Arch Index values between optical pedography and barometric platform.

Parameter	Left Foot	Right Foot
Podoscan AI (mean ± SD)	0.284 ± 0.055	0.286 ± 0.048
Barometry AI (mean ± SD)	0.188 ± 0.092	0.169 ± 0.072
Mean difference (Podo − Baro)	0.097 ± 0.077	0.117 ± 0.056
Paired *t*-test (t)	7.802	12.829
*p*-value	<0.001	<0.001
Pearson r	0.562	0.628
ICC(2,1)	0.494	0.581
Cohen’s d	1.27	2.08
Bland–Altman LoA	−0.053 to 0.247	0.007 to 0.227

**Abbreviations**: AI, Arch Index; SD, standard deviation.

## Data Availability

The data presented in this study are available on reasonable request from the corresponding author due to privacy restrictions.
